# Accurate Learning with Few Atlases (ALFA): an algorithm for MRI neonatal brain extraction and comparison with 11 publicly available methods

**DOI:** 10.1038/srep23470

**Published:** 2016-03-24

**Authors:** Ahmed Serag, Manuel Blesa, Emma J. Moore, Rozalia Pataky, Sarah A. Sparrow, A. G. Wilkinson, Gillian Macnaught, Scott I. Semple, James P. Boardman

**Affiliations:** 1 MRC Centre for Reproductive Health, University of Edinburgh, Edinburgh, UK; 2 Department of Radiology, Royal Hospital for Sick Children, Edinburgh, UK; 3 Clinical Research Imaging Centre, University of Edinburgh, Edinburgh, UK; 4 Centre for Clinical Brain Sciences, University of Edinburgh, Edinburgh, UK

## Abstract

Accurate whole-brain segmentation, or brain extraction, of magnetic resonance imaging (MRI) is a critical first step in most neuroimage analysis pipelines. The majority of brain extraction algorithms have been developed and evaluated for adult data and their validity for neonatal brain extraction, which presents age-specific challenges for this task, has not been established. We developed a novel method for brain extraction of multi-modal neonatal brain MR images, named ALFA (Accurate Learning with Few Atlases). The method uses a new sparsity-based atlas selection strategy that requires a very limited number of atlases ‘uniformly’ distributed in the low-dimensional data space, combined with a machine learning based label fusion technique. The performance of the method for brain extraction from multi-modal data of 50 newborns is evaluated and compared with results obtained using eleven publicly available brain extraction methods. ALFA outperformed the eleven compared methods providing robust and accurate brain extraction results across different modalities. As ALFA can learn from partially labelled datasets, it can be used to segment large-scale datasets efficiently. ALFA could also be applied to other imaging modalities and other stages across the life course.

Magnetic resonance imaging (MRI) is a powerful technique for assessing the brain because it can provide cross-sectional and longitudinal high-resolution images with good soft tissue contrast. It is well-suited to studying brain development in early life, investigating environmental and genetic influences on brain growth during a critical period of development, and to extract biomarkers of long term outcome and neuroprotective treatment effects in the context of high risk events such as preterm birth and birth asphyxia^1^,^2^,^3^,^4^,^5^,^6^,^7^.

Whole-brain segmentation, also known as brain extraction or skull stripping, is the process of segmenting an MR image into brain and non-brain tissues. It is the first step in most neuroimage pipelines including: brain tissue segmentation and volumetric measurement^8^,^9^,^10^,^11^,^12^; template construction^13^,^14^,^15^; longitudinal analysis^16^,^17^,^18^,^19^; and cortical and sub-cortical surface analysis^20^,^21^,^22^,^23^. Accurate brain extraction is critical because under- or over-estimation of brain tissue voxels cannot be salvaged in successive processing steps, which may lead to propagation of error through subsequent analyses.

Several brain extraction methods have been developed and evaluated for adult data. These can be classified into non-learning- and learning-based approaches. Non-learning-based approaches assume a clear separation between brain and non-brain tissues, and no training data are required. For instance, the Brain Extraction Tool (BET) uses a deformable surface model to detect the brain boundaries based on local voxel intensity and surface smoothness^24^, while the Brain Surface Extractor (BSE) methodology combines morphological operation with edge detection^25^. 3dSkullStrip (3DSS) from the AFNI toolkit^26^ is a modified version of BET in order to avoid segmentation of eyes and ventricles and reduce leakage into the skull. The Hybrid Watershed Algorithm^27^ combines watershed segmentation with a deformable-surface model, in which the statistics of the surface curvature and the distance of the surface to the centre of gravity are used to detect and correct inaccuracies in brain extraction.

Learning-based approaches use a set of training data to segment a target or test image. A popular learning-based technique for brain MRI is multi-atlas segmentation^28^,^29^,^30^,^31^, where multiple manually-segmented example images, called atlases, are registered to a target image, and deformed atlas segmentations are combined using label fusion (such as Majority Vote (MV)^28^,^31^, STAPLE^32^ or Shape-based averaging (SBA)^33^; for review see Iglesias and Sabuncu)^34^. The advantage of multi-atlas segmentation methods is that the effect of registration error is minimised by label fusion, which combines the results from all registered atlases into a consensus solution, and this produces very accurate segmentations^34^. In the context of brain extraction: Leung *et al*.^35^ used non-rigid image registration to register best-matched atlas images to the target subject, and the deformed labels were fused using a shape-based averaging technique^33^; Heckemann *et al*.^36^ used an iterative refinement approach to propagate labels from multiple atlases to a given target image using image registration; Doshi *et al*.^37^ used a set of atlas images (selected using K-means) with non-rigid image registration, and a weighted vote strategy was used for label fusion; and Eskildsen *et al*.^38^ proposed a method in which the label of each voxel in the target image is determined by labels of a number of similar patches in the atlas image library. In addition, Brainwash (BW)^39^ uses nonlinear registration from the Automatic Registration Toolbox (ART) with majority vote; and ROBEX^40^ combines a discriminative random forest classifier with a generative point distribution model.

The neonatal brain presents specific challenges to brain extraction algorithms because of: marked intra- and inter-variation in head size and shape in early life; movement artefact; rapid changes in tissue contrast associated with myelination, decreases in brain water, and changes in tissue density; and low contrast to noise ratio between grey matter (GM) and white matter (WM). Most of the methods described above were optimised and evaluated on adult data and their validity for neonatal brain extraction has not been established.

Yamaguchi *et al*.^41^ proposed a method for skull stripping of neonatal MRI, which estimates intensity distributions using *a priori* knowledge based Bayesian classification with Gaussian mixture model, and then a fuzzy rule-based active surface model is used to segment the outer surface of the whole brain. Also, Mahapatra^42^ proposed a neonatal skull stripping technique using prior shape information within a graph cut framework. Recently, Shi *et al*.^43^ developed a framework for brain extraction of paediatric subjects which uses two freely available brain extraction algorithms (BET and BSE) in the form of a meta-algorithm^44^ to produce multiple brain extractions, and a level-set based label fusion is used to combine the multiple candidate extractions together with a closed smooth surface. The methods proposed by Yamaguchi *et al*.^41^ and Shi *et al*.^43^ rely on accurate detection of brain boundaries and have the risk of failing if the algorithm cannot successfully detect the brain boundaries. Also, Mahapatra^42^ and Shi *et al*.^43^ evaluated their methods on T2-weighted (T2w) scans only and their performance on other modalities such as T1-weighetd (T1w) is unknown.

In this article, we present a new method for neonatal whole-brain segmentation from MRI called ALFA (Accurate Learning with Few Atlases), within a multi-atlas segmentation strategy. A typical multi-atlas framework consists of three main components: atlas selection, image registration and label fusion. The proposed method differs from current multi-atlas approaches in the following ways. First, in the atlas selection step, most multi-atlas techniques use a strategy whereby a number of most similar atlas images for each target image is selected^45^. While these strategies can achieve high levels of accuracy, they may be computationally demanding, and lack the scalability to large and growing databases due to limited availability of the large number of manually labelled images on which they depend. In contrast, ALFA eliminates the need for target-specific training data by selecting atlases that are ‘uniformly’ distributed in the low-dimensional data space. This approach also provides information from a range of atlas images, and this benefits learning based label fusion techniques by providing complementary information to the fusion algorithm.

Second, ALFA uses a machine learning voxel-wise classification where a class label for a given testing voxel is determined based on its high-dimensional feature representation. In addition to voxel intensities which are utilised by most of label fusion approaches, we incorporate more information into the features, such as gradient-based features. Figure 1 shows an outline of the proposed method.

We evaluate the method using neonatal T1w and T2w datasets and compare its performance, defined as the agreement between the automatic segmentation and the reference segmentation, with eleven publicly available brain extraction methods that are a representation of a range of learning and non-learning techniques.

## Results

MRI data from 50 preterm infants (mean PMA at birth 29.27 weeks, range 25.43–34.84 weeks) were scanned at term equivalent age (mean PMA 39.64 weeks, range 38.00–42.71 weeks). None of the infants had focal parenchymal cystic lesions.

### Validity of reference segmentations

Ground truth accuracy of reference masks was evaluated by an expert and corrected, when necessary, by a trained rater. The mean (SD) Dice coefficient between corrected and uncorrected segmentations was 89.13 (0.67)%, while the mean (SD) Hausdorff distance was 7.23 (0.96) mm.

To evaluate the reliability of the reference brain masks, we manually segmented the MR images from 10 randomly chosen subjects. The mean (SD) of the Dice coefficient and Hausdorff distance between the reference and manual segmentations of the first rater were 98.61 (0.25)% and 4.94 (1.75) mm, respectively. The mean (SD) of the Dice Coefficient and Hausdorff distance between the reference and manual segmentations of the second rater were 98.03 (0.29)% and 6.62 (1.17) mm, respectively. The inter-rater agreement between the two raters was 98.40 (0.37)%.

### Comparison with other methods and across modalities

The proposed method ALFA was evaluated in comparison with eleven publicly available methods that include non-learning- and learning-based methods: [1] 3dSkullStrip (3DSS) from the AFNI toolkit^26^, [2] BET^24^, [3] BSE^25^, [4] LABEL^43^, [5] ROBEX^40^, [6] Majority Vote (MV)^28^,^31^, [7] STAPLE^32^, [8] Shape-based averaging (SBA)^33^, [9] Brainwash (BW) from the Automatic Registration Toolbox (ART)^39^, [10] MASS^37^, and [11] BEaST^38^. The parameters used for each of these methods were selected as described in Methods.

ALFA produced the highest accuracy among all evaluated methods: average Dice coefficient of 98.94% (T2w) and 97.51% (T1w); average Hausdorff distance of 3.41mm (T2w) and 3.41 mm (T1w); average sensitivity of 98.58% (T2w) and 97.24% (T1w); average specificity of 99.30% (T2w) and 97.78% (T1w). For both T1w and T2w, ALFA’s Dice coefficients were significantly higher when compared to all eleven methods (*P* < 0.05, FDR corrected).

**Figure 2:**
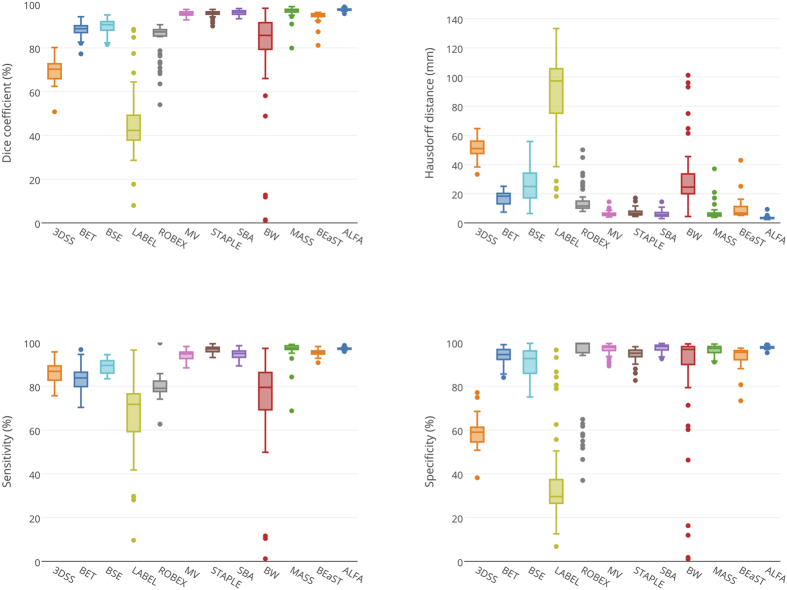
Box plots of Dice coefficient, Hausdorff distance, sensitivity, and specificity for T1w. The plots do not include data from eleven cases when MASS crashed (see Methods).

**Figure 3:**
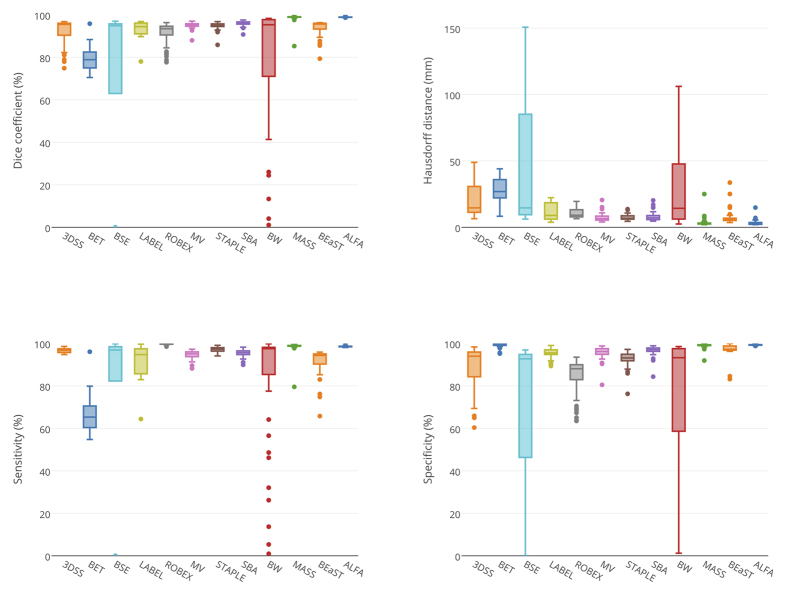
Box plots of Dice coefficient, Hausdorff distance, sensitivity, and specificity for T2w modality.

**Figure 4:**
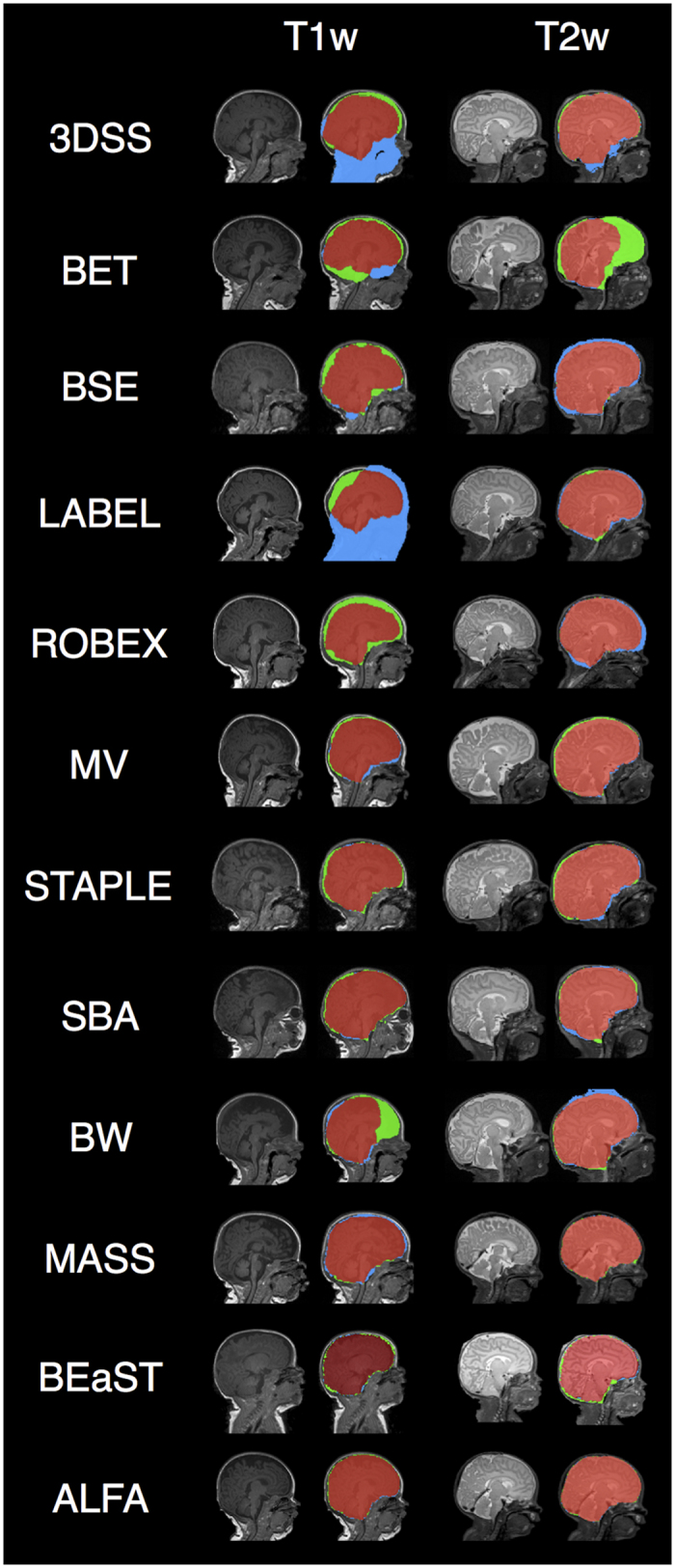
Typical brain extraction results for different methods. The figure shows, for each method, the case with median Dice coefficient for T1w and T2w. Green: reference segmentation; Blue: automatic; Red: overlap between reference segmentation and automatic segmentation.

Figures 2 and 3 show box plots with different metrics values for the evaluated methods on the T1w and T2w modalities, and Table 1 shows means and standard deviations (SD) of the evaluation metrics for both modalities. Figure 4 shows sample outputs, i.e. the case with median Dice coefficient, from each method. For presented ALFA results, *k* = 3 for both image sequences.

### Localisation of segmentation error

**Figure 5:**
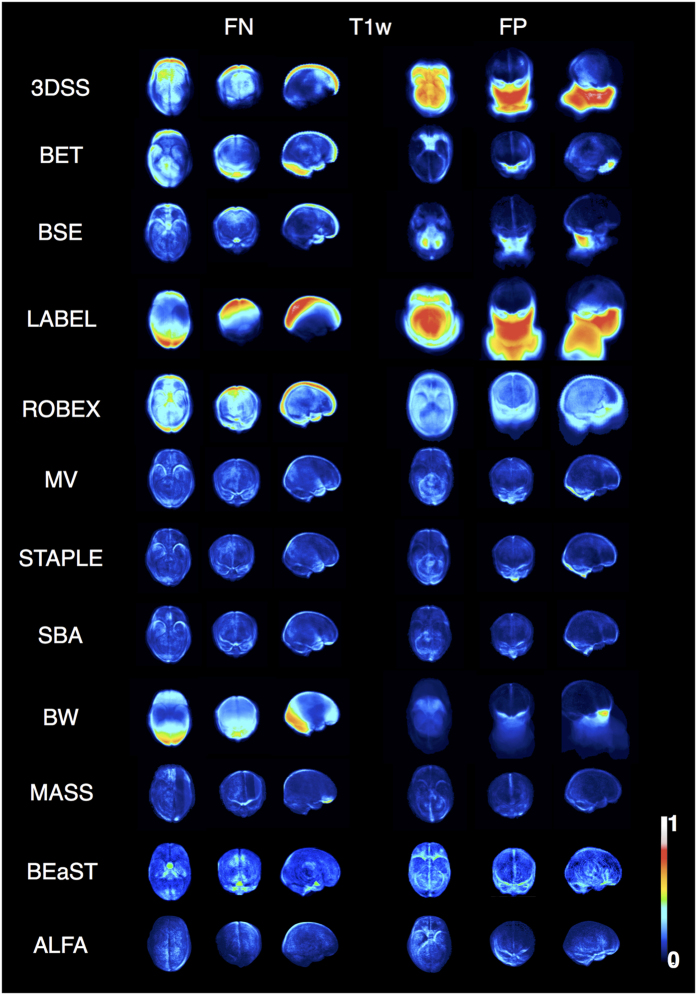
Axial, coronal, and sagittal projections of the false-negative (FN) and false-positive (FP) spatial probability maps for the different methods for T1w. The maps are scaled from 0 to 1.

**Figure 6:**
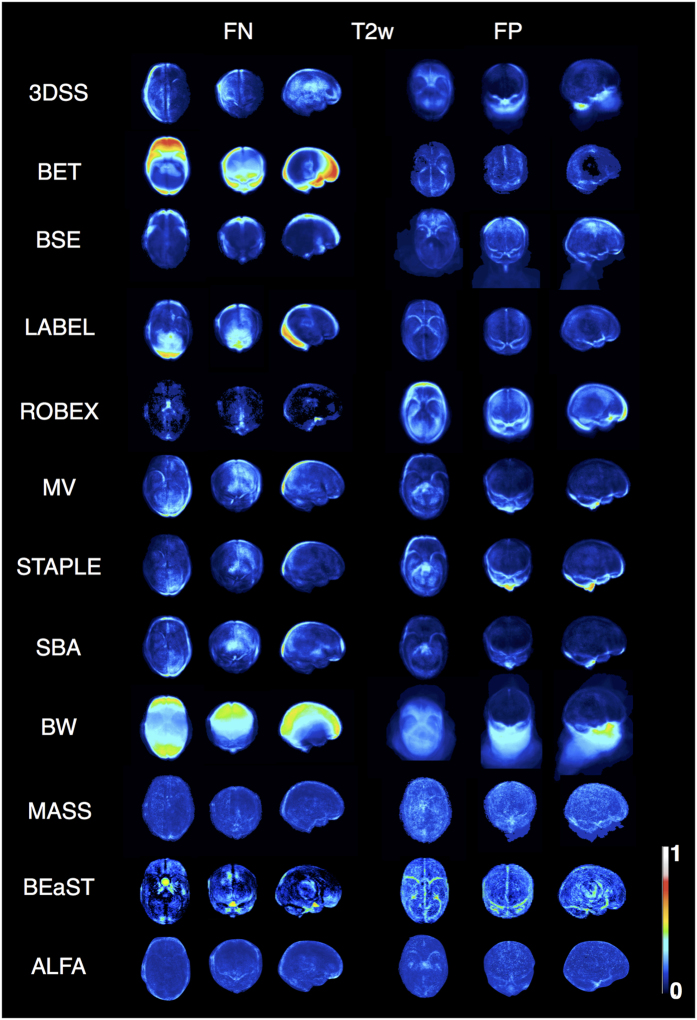
Axial, coronal, and sagittal projections of the false-negative (FN) and false-positive (FP) spatial probability maps for the different methods for T2w. The maps are scaled from 0 to 1.

Projection maps display average error in anatomic space for each algorithm (Figs 5 and 6). ALFA’s noticeable error was leaving in tissue along the borders of the temporal lobe, and leaving out tissue along the border of the parietal and occipital lobes, however ALFA’s rate of false positives and false negatives was noticeably less than the other methods. Other common errors included non-learning based methods (3DSS, BET, BSE) leaving in extra neck tissue and/or eye; learning-based methods (MV, STAPLE, SBA) over-segmenting the cerebellum and the bottom of the brainstem (T2w), while under-segmenting the parietal lobe; BW leaving in neck tissue and eye; ROBEX over-segmenting the cerebellum and the temporal (T1w), frontal, occipital and parietal lobes (T2w); LABEL leaving in neck tissue and eye (T1w), while under-segmenting the occipital lobe; BEaST under-segmenting the brainstem, occipital and frontal (T2w) lobes, while over-segmenting the cerebellum, frontal and parietal (T1w) lobes; MASS leaving out tissue along the border of the frontal lobe close to the eye (T1w), while leaving in tissue in the occipital lobe (T1w).

### Evaluating the feature importance and classifier performance

**Figure 7:**
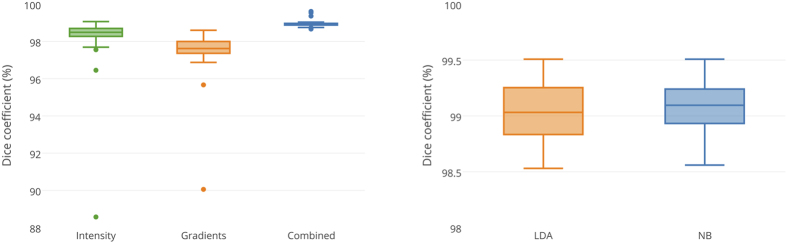
Feature importance (Intensity, Gradients, and Combined) [left], and classifier performance (Linear Discriminant Analysis [LDA] and Naïve Bayes [NB]) [right].

We used two main categories of features: intensity features and gradient-based features. Figure 7 shows that intensity features alone provided higher accuracy than gradient-based features. However, combining both categories yielded higher accuracy than each individual category (*P* < 0.001). We tested two different linear classification techniques: Linear Discriminant Analysis (LDA) and Naïve Bayes (NB) demonstrated equivalent performance, with both providing a very high accuracy.

### Evaluating the effect of atlas selection strategy on ALFA’s performance

**Figure 8:**
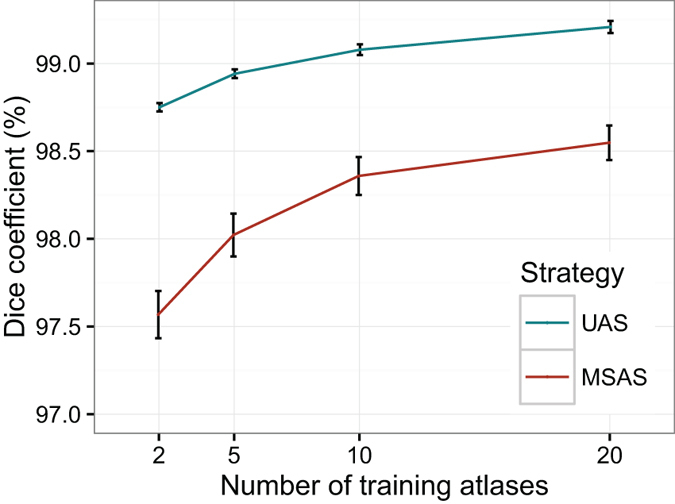
ALFA performance using different atlas selection strategies. Most Similar Atlas Selection (MSAS) and Uniform Atlas Selection (UAS).

We compared an atlas selection strategy based on the number of most similar atlases to the target subject (MSAS), with the proposed strategy of using uniformly distributed data (UAS). Although Fig. 8 shows that accuracy increases with higher numbers of training atlases, the segmentation accuracy of UAS does not benefit greatly from an increase in number of atlases as Dice coefficient only increases by < 0.5% [from 98.8% (*k* = 2) to 99.2% (*k* = 20)]. When using MSAS strategy, the segmentation accuracy increases from 97.6% (*k* = 2) to 98.5% (*k* = 20) [almost 1% increase]. In addition, using a set of two training atlases that are selected with UAS strategy provides greater accuracy than twenty atlases selected using the MSAS strategy.

### Volume measurement

**Figure 9:**
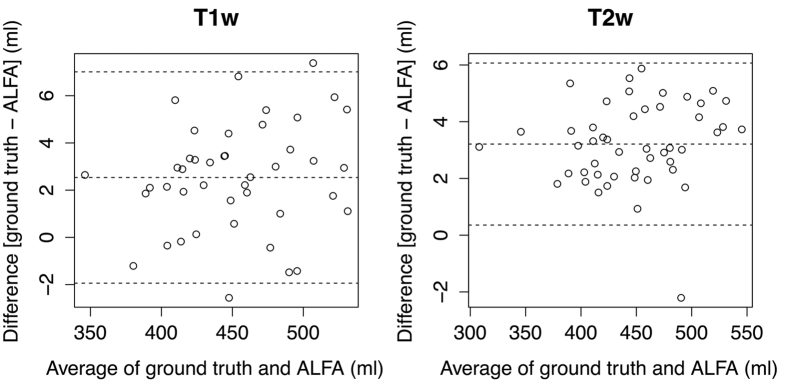
A Bland-Altman plot showing the agreement between volume measurement based on reference and automatic segmentations of the neonatal brain for T1w and T2w. The middle line represents the mean and the outer lines represent ±1.96 standard deviations.

To evaluate the utility of ALFA for extracting whole brain volume from T1w and T2w datasets, we measured agreement between volumes derived from ALFA with reference values for both modalities. Figure 9 shows that ALFA provides a level of agreement that is likely to be acceptable for most clinical and experimental applications. There was no statistically significant difference between mean brain volumes estimated from T1w and T2w datasets (mean difference = 4.12 ml, *P* = 0.25); the difference observed may reflect differences in the masks created from the original templates.

### Computation time

The experiments for 3DSS, BET, BSE, LABEL, ROBEX, BW, BEaST and MASS were run on a 64-bit Linux machine (Intel^®^ Xeon^®^ CPU E5-2650 @ 2.00 GHz x 18, 64 GB RAM), and the experiments for MV, STAPLE, SBA and ALFA were run on a 64-bit iMac^®^ (Intel^®^ Core i7 @ 3.5 GHz × 4, 32 GB RAM). 3DSS, BSE and ROBEX methods take less than a minute to perform a single brain extraction. BET (with chosen parameters for neck and eye cleanup) takes ~8 minutes. LABEL takes ~3 minutes to complete a single brain extraction. As BW, MASS, MV, STAPLE, SBA and ALFA are multi-atlas-based methods, the computation time of a single extraction is a combination of two processes: registration and fusion. A single registration of BW or BEaST, takes ~3 minutes; a single registration of MASS, based on DRAMMS registration framework^46^, takes ~20 minutes; and a single registration of MV, STAPLE, SBA or ALFA takes ~5 minutes (less than a minute based on an free-form registration using graphic processing unit^47^). The fusion time for all the multi-atlas based approaches (including ALFA) takes less than a minute.

## Discussion

In this article, we propose a new method (Accurate Labeling with Few atlases, ALFA) for brain extraction of neonatal MRI and demonstrate that it provides robust and accurate results for T1w and T2w neonatal data. The method belongs to the multi-atlas family where a number of training atlases are used to train a voxel-wise local classifier. The atlas selection strategy of ALFA has a crucial role because the use of a number of atlases that are ‘uniformly’ distributed in the low-dimensional data space provides information from a range of images and this benefits the classification process. The method contrasts with atlas selection strategies that select the most similar atlases to the test subject and hence provide less complementary information to the algorithm^45^. Also, the most similar atlas selection strategy is best suitable for large databases of images where for each subject, a large number of similar subjects (*k* ≥ 20) exists^35^,^45^. With ALFA, atlases with relatively large anatomical variability could be selected but this does not represent a problem because the algorithm requires alignment of global brain boundaries and not local structures. While alternative approaches for image registration with large anatomical variation could be used^19^,^48^, this would be at the expense of computation time.

As ALFA employs a sparsity-based technique to select a set of representative atlases from the target dataset, it eliminates the need for target-specific training data; quite similar to MASS^37^. However MASS uses K-means to cluster the images, with subsequent selection of a number of images from each cluster, and K-means can fail when clusters of arbitrary shapes are present in the data because of sub-optimal selection of representative images and neglect of some clusters^49^. It is worth mentioning that there are other sparsity- and label-propagation-based techniques of interest that were applied to a range of medical image segmentation problems such as prostate segmentation from CT images^50^, hippocampus labeling in adult MRI^51^,^52^, and brain tissue segmentation and structural parcellation^53^.

In our leave-one-out cross-validation, learning-based approaches outperformed the non-learning-based methods. 3DSS, BET and BSE performed less well in extracting the neonatal brain compared to their established performance on adult data^40^. LABEL, which was designed and evaluated for paediatric and neonatal data, provided an acceptable accuracy on T2w (average Dice coefficient of 93.54%), however it did not perform well with respect to other methods on T1w data (average Dice coefficient of 45.62%). MASS outperformed MV, STAPLE, SBA (which are considered the benchmark for learning-based approaches); however ALFA provided accurate and robust results across modalities compared to MASS as well as the benchmark methods. It is notable that MASS crashed in eleven T1w cases (more details in Methods), and it takes ~20 minutes for a single registration. As the learning-based approaches are trained using the same set of selected atlases, the performance difference between the methods is a function of the accuracy of the registration algorithm used and/or the label fusion strategy (comparison of different registration approaches and label fusion schemes can be found in Klein *et al*.^54^, and Iglesias and Sabuncu^34^).

ROBEX is a special case in our comparison since it combines generative and discriminative approaches. It is similar to ALFA in that it uses voxel-wise classification to refine the voxels at brain boundaries, but the major difference between the two is that ROBEX uses an adult template as standard space for training the voxel-wise classifier, and where the target subject is supposed to be aligned. This limits the flexibility of ROBEX to work with different imaging modalities and young populations. In contrast to ROBEX, ALFA just needs a small number of manually labelled images from the population under study to provide very accurate results. Typically, 2–5 training images are sufficient, however this need might increase depending on the morphological variation within the population under study. Another important difference is that ROBEX uses a global classifier which uses the voxel coordinates as features (beside other features), but ALFA uses a local classifier which is trained by information from the neighbouring voxels so it is less susceptible to classification errors.

Regarding the performance of the compared methods across modalities, the eleven methods provided better performance on T2w images compared with T1w images. This might be because the T2w images have better contrast than the T1w images and hence the brain boundaries can be detected more accurately on T2w images comparing to T1w. Also the better contrast on T2w images means that the registration process for learning-based methods is more accurate. It is worth mentioning also that evaluating the performance of the proposed method on different datasets was not performed because the main idea behind this work is to be able to provide accurate segmentation results using a very small number of within-study training images (which is not a labour intensive process), instead of the commonly used strategy of selecting training images from an external library.

We used a semi-automatic approach (automatic segmentations that were manually edited by a rater) to generate the reference brain masks. We chose this approach partly because of its accuracy in a recent evaluation of automatic neonatal brain segmentation algorithms^55^, and partly because it is more time-efficient than mask generation from scratch. It is possible that ALFA (in common with all other learning-based methods) may have an advantage over non-learning-based methods in the comparison because the reference segmentations were generated via a learning-based framework. However, any advantage conferred to learning-based methods is likely to be minimal for the following reasons. First, validation of the reference masks against a subset of manually delineated masks showed a very high agreement between reference and manually delineated masks. Second, ALFA and the learning-based methods show variable accuracies as the false positive rate and false negative rate maps of the learning-based methods show errors in various anatomical regions. This suggests that there is still inconsistency between the segmentations from learning based methods (including ALFA) and reference segmentations.

A possible limitation is that we tuned the parameters of 3DSS, BET and BSE based on previous experience^1^,^8^,^14^,^20^, and the suggestions from the authors of the methods, but it is possible that an expert user may be able to optimise parameters to produce improved results. Also, MV, STAPLE, SBA, BEaST, and BW might yield better results using an increased number of training atlases and/or a different atlas selection strategy. However, our intention was to design a method that can provide accurate results using a relatively small number of training data, and this formed the basis of the comparison study. It is worth mentioning that engines such as Segmentation Validation Engine^56^ would be ideal for evaluating the performance of the different methods for adult brain data.

To conclude, we present a novel method for extracting neonatal brain MRI that is robust and provides accurate and consistent results across modalities, which is useful because T1w and T2w data enable different yet complementary inferences about developmental processes. As ALFA can learn from partially labelled datasets, it can be used to segment large-scale datasets efficiently. Although ALFA was implemented and evaluated on neonatal MR images, the idea is generic and could be applied to other imaging modalities and other stages of the life course. ALFA is available to the research community at http://brainsquare.org.

## Methods

### Ethical Statement

Ethical approval was obtained from the National Research Ethics Service (South East Scotland Research Ethics Committee), and informed written parental consent was obtained. The methods were carried out in accordance with the approved guidelines.

### Participants

Preterm infants were recruited prospectively from the Royal Infirmary of Edinburgh between July 2012 and January 2015. Inclusion criteria: birthweight <1500 g or postmenstrual age (PMA) <33 weeks’ gestation. Exclusion criteria: major congenital malformations; chromosomal disorders; congenital infection; and infants with cystic periventricular leucomalacia, hemorrhagic parenchymal infarction or post-hemorrhagic ventricular dilatation detected on cranial ultrasound or MRI. Infants were scanned during natural sleep.

### Image acquisition

A Siemens Magnetom Verio 3T MRI clinical scanner (Siemens AG, Healthcare Sector, Erlangen, Germany) and 12-channel phased-array head coil were used to acquire: (1) T1-weighted 3D MPRAGE: TR = 1650 ms, TE = 2.43 ms, inversion time = 160 ms, flip angle = 9°, acquisition plane = sagittal, voxel size = 1 × 1 × 1 mm^3^, FOV = 256 mm, acquired matrix = 256 × 256, acquisition time = 7 min 49 sec, acceleration factor (iPAT) = 2; (2) T2-weighted SPACE: TR = 3800 ms, TE = 194 ms, flip angle = 120°, acquisition plane = sagittal, voxel size = 0.9 × 0.9 × 0.9 mm^3^, FOV = 220 mm, acquired matrix = 256 × 218, acquisition time = 4 min 34 sec. The image data used in this manuscript are available from the BRAINS repository^57^ (http://www.brainsimagebank.ac.uk).

### Preprocessing

Images were corrected for intensity inhomogeneity using the N4 method^58^, and reconstructed to isotropic voxel size (1 × 1 × 1 mm^3^) using windowed sinc interpolation.

### Reference brain masks and atlas library construction

The reference brain masks of the atlas library that was used for training, validation and method comparison was created using the following approach. First, all the images from the dataset were nonlinearly aligned to the 40 weeks PMA template from the 4D atlas constructed in Serag *et al*.^14^. Then, an Expectation–Maximization framework for brain tissue segmentation (defined as white matter, grey matter and cerebrospinal fluid) was used, where the priors were propagated using prior probabilities provided by the 4D atlas. Finally, brain masks were deformed to the subjects’ native space. Generated masks were inspected for accuracy by a radiologist experienced in neonatal brain MRI, and edited by a trained rater, when necessary.

To evaluate the reliability of the reference brain masks, an independent rater segmented the MR images from 10 randomly chosen subjects (5 T1w and 5 T2w) using ITK-SNAP (http://itksnap.org) to separate brain (grey and white matter, and cerebrospinal fluid) and non-brain voxels (such as skull, eye and optic nerve). Similarly, to assess the inter-rater variability, a different rater delineated the brains from the same 10 images.

### Atlas selection

**Figure 10:**
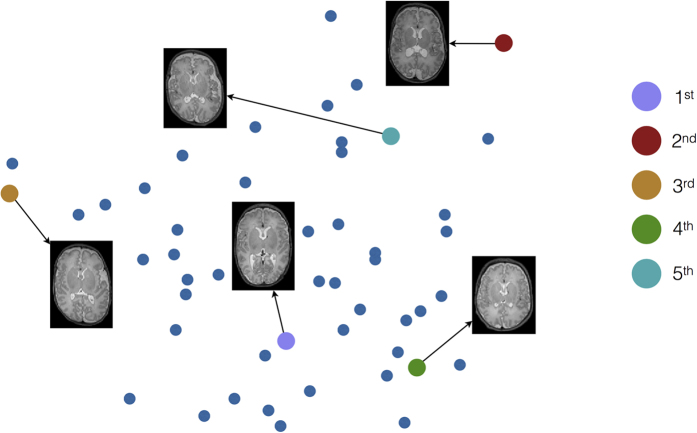
Illustration of the atlas selection principle. The brain images represent five chosen atlases, and the colour codes represent the order these atlases were chosen.

In this work, we use a sparsity-based technique to select a number of representative atlas images that capture population variability by determining a subset of *n*-dimensional samples that are ‘uniformly’ distributed in the low-dimensional data space. Let 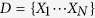 be a set of training images from *N* subjects. To select a subset *S* of *k* images where *k* ≤ *N* (optimally, *k* ≪ *N*), the atlas selection algorithm works as follows. First, all images from the training dataset are linearly registered (12 degrees of freedom) to the 40 weeks PMA template from the 4D atlas^14^, which is the closest age-matched template to the mean age of the subjects in the training dataset, and image intensities are normalised using the method described by Nyul and Udupa^59^. Then, all *N* aligned images are considered as candidates for the subset of selected atlases. The closest image to the mean of the dataset is included as the first subset image. Let us refer to it as *S*_1_. The consecutive images are selected sequentially, based on the distances to the images already assigned to the subset. The distance from the *i*-th to the *j*-th image, *d*(*i, j*) is defined as:


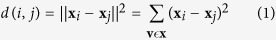


where 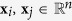 are data vectors obtained by concatenating the voxel, *v*, values of *X*_*i*_, *Y*_*j*_, respectively. The main steps of the proposed atlas selection algorithm are presented in Algorithm 1, and Fig. 10 shows an illustration of the atlas selection principle.

It is worth mentioning that the proposed atlas selection strategy was inspired by the *Kennard-Stone* algorithm^60^, yet different in the way it is initialised. The *Kennard-Stone* algorithm begins by finding the two images which are farthest apart, however the proposed algorithm begins by finding the closest image to the mean of the dataset.

### Image registration

Image registration was carried out in two steps: first, a linear transformation was estimated using affine registration (12 degrees of freedom); second, a nonlinear registration step was carried out using the result of the affine registration as the initial transformation. The registration scheme is based on free-form deformations (FFD)^47^,^61^ with normalised mutual information as the similarity metric^62^. The nonlinear registration was carried out in a coarse-to-fine manner with successive control point spacing of 20 mm, 10 mm, and 5 mm. All registration steps were carried out using the open-source image registration toolkit NiftyReg (https://sourceforge.net/projects/niftyreg), using default settings.

**Algorithm 1.** Uniform atlas selection algorithm

**Input:** 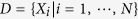


**Output:** 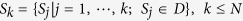


Set **x** to represent the vector obtained by concatenating the voxel, *v*, values of an image *X*;

Set 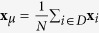 to be the vector which represents the mean *μ* of the dataset;

Set *m* to represent the number of currently selected images;

**foreach** *i* ∈ *D* **do**


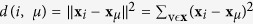


**end**

Select 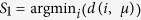;

Increase *m* by 1;

**while** *m* < *k* **do**

**  foreach** *i* ∉ *S* **do**


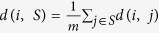


** end**

 Select 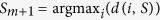;

 Increase *m* by 1;

**end**

### Label fusion

Machine learning was used to assign a label to each voxel in the target image. The method is based on training a local classifier for each voxel. In addition to voxel intensities, which are utilised by most of label fusion approaches, we incorporate information from gradient-based features. Typically, each voxel *v* at the location (*x, y, z*) is converted to a five-dimensional feature vector


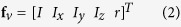


where *I* is the grey scale intensity value, *I*_*x*_, *I*_*y*_ and *I*_*z*_ are the absolute norms of the first order derivatives with respect to *x, y* and *z*, and the gradient magnitude *r* is defined as 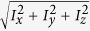. The image derivatives are calculated through the filter [−1 0 1]^*T*^. The vector in equation (2) represents the testing sample. The training samples come from the deformed atlas images where feature vectors are extracted in the deformed atlas images from the 26-adjacent voxels, which means that the number of training samples per voxel is equal to *k* × 26.

Finally, linear discrimination techniques [such as Naïve Bayes (NB) and Linear Discriminant Analysis (LDA)] were used to classify the target image voxels into brain or non-brain.

### Compared methods, parameter selection and software considerations

The compared methods are listed in Table 2, and the parameter setting were determined as follows:

**3DSS**^26^. The parameters used for 3DSS: –shrink_fac_bot_lim 0.65,–shrink_fac 0.72 (suggested by the AFNI team). No problems encountered during running the software.

**BET**^24^. The parameters used for BET: -f 0.5, -g −0.1, -R, -B. These parameters were set based on our experience using BET in previous neonatal studies^1^,^8^,^14^,^20^. No problems encountered during running the software.

**BSE**^25^. The parameters used for BSE: -d 20, -r 2, -s 0.8, -n 3, -p, –trim. We reached these setting by trying the interactive version of the software and experiment with the parameters there (as suggested by the BSE authors). No problems encountered during running the software.

**LABEL**^43^. No parameters needed, and no problems encountered during running the software.

**ROBEX**^40^. No parameters needed, and no problems encountered during running the software.

**MV**^28^,^31^. Leung and colleagues^35^ showed that the accuracy of different label fusion techniques (MV, STAPLE, and SBA) for whole-brain segmentation started to reach a plateau when combining more than 19 segmentations. Based on that, for each test subject, we choose *k* = 20 most similar atlas images. No problems encountered during running the software.

**STAPLE**^32^. *k* = 20 atlases (see above). No problems encountered during running the software.

**SBA**^33^. *k* = 20 atlases (see above). No problems encountered during running the software.

**BW**^39^. Similar to MV, BW adopts a majority vote strategy. Hence, we chose to set *k* to 20 atlases. No problems encountered during running the software.

**MASS**^37^. *k* = 5 atlases were selected as in Doshi *et al*.^37^. Several crashes were encountered for T1w and T2w cases, and in the first run of the software only 10 T2w and 6 T1w were complete. By sharing the issue with the authors, we were advised to re-run the software as the issue might be because one or more registration jobs have failed to finish or was killed prematurely. After listening to the authors advice and re-running the software several times on the failed subjects, the whole T2W cases were complete (after 3 additional runs), however 11 T1w cases were just very stubborn to complete (even after all the re-runs we tried).

**BEaST**^38^. *k* = 20 atlases were selected as suggested in Eskildsen *et al*.^38^. Neonatal reference brain scans were used inside of the BEaST framework, replacing adult training dataset. No problems encountered during running the software.

### Validation framework

A leave-one-out cross-validation procedure was performed for the 50 subjects. Each subject in turn was left out as a testing sample and the remaining 49 subjects were used as the training dataset where a subset of *k* atlases is selected using Algorithm 1. Agreement between the automatically segmented brain mask *A* and the reference mask *M* was evaluated using two complementary overlap metrics:

#### Dice coefficient

The Dice coefficient *D*^63^ measures the extent of spatial overlap between two binary images. It ranges between 0 (no overlap) and 1 (perfect agreement). The Dice values are obtained using equation (3) and expressed as a percentage.


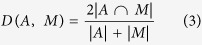


#### Hausdorff distance

The Hausdorff distance *H* is generally used to measure the spatial consistency of the overlap between the two binary images by measuring the maximum surface-to-surface distance between the two images. It is given by


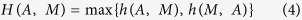


where


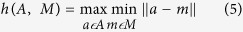


In addition to the overlap agreement measures, we compute sensitivity, defined as


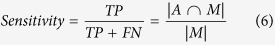


and specificity, defined as


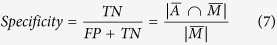


where *TP* is the true positive, *TN* is the true negative, *FP* is the false positive, *FN* is the false negative, and |·| is the number of elements in a set. Similar to the Dice values, both sensitivity and specificity are expressed as a percentage.

### Localisation of segmentation error

Projection maps were generated for false positive and false negative voxels, which enables the localisation of segmentation errors. First, the false positive/negative maps were aligned to one coordinate space, before averaging and projecting onto axial, coronal and axial orientations. For each method, and each modality, the projection maps give insight into the spatial locations where major false positive/negative voxels exist, and hence it can be used to compare the performance of the evaluated methods against each other and across modalities.

### Statistical analyses

To test for differences between the results of the methods, t-tests were used for normally distributed data, and Mann Whitney *U* was used to compare non-normal distributions (Shapiro-Wilk normality test was used). *P*-values < 0.05 were considered significant after controlling for Type I error using false discovery rate (FDR).

The effect of feature importance, classifier performance and atlas selection was evaluated based on T2w images using the Dice coefficient. Note that to evaluate the effect of atlas selection strategy on the performance, the most similar atlas selection strategy (MSAS) was implemented as explained in the atlas selection section with one difference. This difference is that instead of aligning all images to a template, all images were aligned to the test image and the distance between warped training images and the test image was calculated using equation (1). Then, the most similar *k* atlases were selected.

Agreement between whole brain volumes extracted from T1w and T2w images using ALFA compared to the reference segmentation was investigated using Bland-Altman methods.

## Additional Information

**How to cite this article**: Serag, A. *et al*. Accurate Learning with Few Atlases (ALFA): an algorithm for MRI neonatal brain extraction and comparison with 11 publicly available methods. *Sci. Rep.* **6**, 23470; doi: 10.1038/srep23470 (2016).

## Figures and Tables

**Figure 1:**
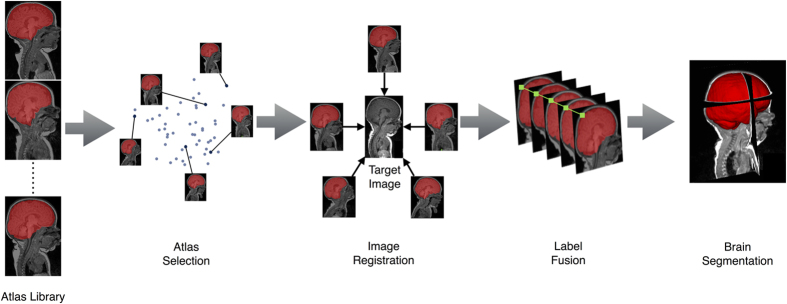
Outline of the proposed method, ALFA. A number of atlas images are selected from the atlas images library and registered to the target image. Then, atlas segmentations are deformed to the target image, and machine learning based label fusion is used to obtain the final brain segmentation.

**Table 1:** Means and standard deviations (SD) of the evaluation metrics (Dice coefficient *D*, Hausdorff distance *H*, Sensitivity *SEN*, Specificity *SPE*) for T1w and T2w images.

	**T1w [Mean (SD)]**				**T2w [Mean (SD)]**			
	***D*** **(%)**	***H (mm)***	***SEN (%)***	***SPE (%)***	***D (%)***	***H (mm)***	***SEN (%)***	***SPE (%)***
3DSS	69.78 (5.27)	51.41 (6.34)	86.46 (4.47)	58.73 (6.42)	92.21 (5.93)	20.28 (12.93)	96.72 (1.05)	88.80 (10.47)
BET	88.36 (3.27)	16.90 (4.66)	83.48 (5.61)	94.17 (3.32)	79.18 (4.95)	28.36 (8.58)	66.21 (7.35)	99.19 (0.92)
BSE	89.62 (3.44)	27.32 (13.83)	89.03 (3.16)	90.62 (6.99)	71.44 (40.83)	38.51 (46.47)	72.99 (41.62)	70.41 (40.55)
LABEL	45.62 (15.59)	86.63 (29.63)	67.63 (16.98)	37.81 (20.69)	93.54 (3.32)	11.92 (6.39)	92.06 (7.03)	95.49 (2.08)
ROBEX	84.07 (7.80)	15.39 (9.22)	82.65 (9.32)	90.34 (18.32)	91.01 (5.53)	10.48 (3.78)	99.76 (0.24)	84.12 (8.89)
MV	95.50 (1.19)	6.09 (1.78)	94.12 (2.23)	97.01 (2.34)	95.11 (1.40)	7.26 (2.98)	94.69 (1.94)	95.63 (2.93)
STAPLE	95.62 (1.47)	7.21 (2.56)	96.83 (1.58)	94.53 (3.15)	94.85 (1.69)	7.35 (2.03)	97.13 (1.18)	92.77 (3.43)
SBA	96.09 (1.11)	6.01 (2.04)	94.67 (2.21)	97.61 (1.79)	96.01 (1.15)	8.00 (3.32)	95.49 (1.64)	96.60 (2.32)
BW	78.83 (23.81)	30.48 (22.91)	73.77 (23.31)	85.69 (25.72)	77.41 (30.31)	29.68 (30.16)	81.74 (29.47)	74.45 (31.42)
MASS	96.50 (3.06)	7.28 (5.97)	96.48 (5.18)	96.69 (1.96)	98.74 (1.96)	3.52 (3.31)	98.50 (2.75)	99.00 (1.12)
BEaST	94.33 (2.71)	9.36 (7.01)	95.38 (1.42)	93.43 (4.81)	93.86 (3.80)	7.61 (5.98)	91.30 (6.76)	97.02 (3.41)
ALFA	97.51 (0.54)	3.40 (1.13)	97.24 (0.51)	97.78 (0.66)	98.94 (0.17)	3.40 (2.10)	98.58 (0.24)	99.30 (0.21)

**Table 2:** Compared methods with their corresponding addresses.

**Method**	**Web address**
ALFA	http://brainsquare.org
3dSkullStrip (3DSS)	http://afni.nimh.nih.gov
BET	http://fsl.fmrib.ox.ac.uk
BSE	http://brainsuite.org
LABEL	http://www.nitrc.org/projects/ibeat
ROBEX	http://www.nitrc.org/projects/robex
Majority Vote (MV)	https://github.com/BioMedIA/IRTK
STAPLE	http://www.itksnap.org/c3d
Shape-based Averaging (SBA)	http://www.nitrc.org/projects/cmtk
Brainwash (BW)	http://www.nitrc.org/projects/art
MASS	http://www.cbica.upenn.edu/sbia/software
BEaST	https://www.mcgill.ca/bic/software/tools-data-analysis/anatomical-mri/beast
